# Supplementary material for: Gene Expression Signature of Acquired Chemoresistance in Neuroblastoma Cells
Source: Int J Mol Sci. 2020 Sep 16;21(18):6811. doi: 10.3390/ijms21186811 (PMC7555742; doi:10.3390/ijms21186811)
Supplement: Supplementary file 1 [file ijms-21-06811-s001.pdf]

Gene table: RT² Profiler™ PCR Array Human Cancer Drug Resistance

(Cat. No. 330231, PAHS-012ZA)

Gene table: RT² Profiler PCR Array

Position	UniGene	GenBank	Symbol	Description
A01	Hs.489033	NM_000927	ABCB1	ATP-binding cassette, sub-family B (MDR/TAP), member 1
A02	Hs.709181	NM_004996	ABCC1	ATP-binding cassette, sub-family C (CFTR/MRP), member 1
A03	Hs.368243	NM_000392	ABCC2	ATP-binding cassette, sub-family C (CFTR/MRP), member 2
A04	Hs.463421	NM_003786	ABCC3	ATP-binding cassette, sub-family C (CFTR/MRP), member 3
A05	Hs.728765	NM_005688	ABCC5	ATP-binding cassette, sub-family C (CFTR/MRP), member 5
A06	Hs.480218	NM_004827	ABCG2	ATP-binding cassette, sub-family G (WHITE), member 2
A07	Hs.171189	NM_001621	AHR	Aryl hydrocarbon receptor
A08	Hs.729170	NM_001283	AP1S1	Adaptor-related protein complex 1, sigma 1 subunit
A09	Hs.158932	NM_000038	APC	Adenomatous polyposis coli
A10	Hs.496240	NM_000044	AR	Androgen receptor
A11	Hs.632446	NM_001668	ARNT	Aryl hydrocarbon receptor nuclear translocator
A12	Hs.367437	NM_000051	ATM	Ataxia telangiectasia mutated
B01	Hs.624291	NM_004324	BAX	BCL2-associated X protein
B02	Hs.150749	NM_000633	BCL2	B-cell CLL/lymphoma 2
B03	Hs.516966	NM_138578	BCL2L1	BCL2-like 1
B04	Hs.371914	NM_000386	BLMH	Bleomycin hydrolase
B05	Hs.194143	NM_007294	BRCA1	Breast cancer 1, early onset
B06	Hs.34012	NM_000059	BRCA2	Breast cancer 2, early onset
B07	Hs.523852	NM_053056	CCND1	Cyclin D1
B08	Hs.244723	NM_001238	CCNE1	Cyclin E1
B09	Hs.19192	NM_001798	CDK2	Cyclin-dependent kinase 2
B10	Hs.95577	NM_000075	CDK4	Cyclin-dependent kinase 4
B11	Hs.370771	NM_000389	CDKN1A	Cyclin-dependent kinase inhibitor 1A (p21, Cip1)
B12	Hs.238990	NM_004064	CDKN1B	Cyclin-dependent kinase inhibitor 1B (p27, Kip1)
C01	Hs.512599	NM_000077	CDKN2A	Cyclin-dependent kinase inhibitor 2A (melanoma, p16, inhibits CDK4)
C02	Hs.435051	NM_001800	CDKN2D	Cyclin-dependent kinase inhibitor 2D (p19, inhibits CDK4)
C03	Hs.444673	NM_030782	CLPTM1L	CLPTM1-like
C04	Hs.72912	NM_000499	CYP1A1	Cytochrome P450, family 1, subfamily A, polypeptide 1
C05	Hs.1361	NM_000761	CYP1A2	Cytochrome P450, family 1, subfamily A, polypeptide 2
C06	Hs.1360	NM_000767	CYP2B6	Cytochrome P450, family 2, subfamily B, polypeptide 6
C07	Hs.282409	NM_000769	CYP2C19	Cytochrome P450, family 2, subfamily C, polypeptide 19
C08	Hs.709188	NM_000770	CYP2C8	Cytochrome P450, family 2, subfamily C, polypeptide 8
C09	Hs.282624	NM_000771	CYP2C9	Cytochrome P450, family 2, subfamily C, polypeptide 9
C10	Hs.648256	NM_000106	CYP2D6	Cytochrome P450, family 2, subfamily D, polypeptide 6
C11	Hs.12907	NM_000773	CYP2E1	Cytochrome P450, family 2, subfamily E, polypeptide 1
C12	Hs.654391	NM_017460	CYP3A4	Cytochrome P450, family 3, subfamily A, polypeptide 4
D01	Hs.695915	NM_000777	CYP3A5	Cytochrome P450, family 3, subfamily A, polypeptide 5
D02	Hs.592364	NM_000791	DHFR	Dihydrofolate reductase
D03	Hs.488293	NM_005228	EGFR	Epidermal growth factor receptor
D04	Hs.181128	NM_005229	ELK1	ELK1, member of ETS oncogene family
D05	Hs.89649	NM_000120	EPHX1	Epoxide hydrolase 1, microsomal (xenobiotic)
D06	Hs.446352	NM_004448	ERBB2	V-erb-b2 erythroblastic leukemia viral oncogene homolog 2, neuro/glioblastoma derived oncogene homolog (avian)
D07	Hs.118681	NM_001982	ERBB3	V-erb-b2 erythroblastic leukemia viral oncogene homolog 3 (avian)
D08	Hs.390729	NM_005235	ERBB4	V-erb-a erythroblastic leukemia viral oncogene homolog 4 (avian)

Position	UniGene	GenBank	Symbol	Description
D09	Hs.469872	NM_000122	ERCC3	Excision repair cross-complementing rodent repair deficiency, complementation group 3 (xeroderma pigmentosum group B complementing)
D10	Hs.208124	NM_000125	ESR1	Estrogen receptor 1
D11	Hs.729020	NM_001437	ESR2	Estrogen receptor 2 (ER beta)
D12	Hs.284244	NM_002006	FGF2	Fibroblast growth factor 2 (basic)
E01	Hs.728789	NM_005252	FOS	FBJ murine osteosarcoma viral oncogene homolog
E02	Hs.466828	NM_019884	GSK3A	Glycogen synthase kinase 3 alpha
E03	Hs.523836	NM_000852	GSTP1	Glutathione S-transferase pi 1
E04	Hs.597216	NM_001530	HIF1A	Hypoxia inducible factor 1, alpha subunit (basic helix-loop-helix transcription factor)
E05	Hs.643120	NM_000875	IGF1R	Insulin-like growth factor 1 receptor
E06	Hs.487062	NM_000876	IGF2R	Insulin-like growth factor 2 receptor
E07	Hs.132966	NM_000245	MET	Met proto-oncogene (hepatocyte growth factor receptor)
E08	Hs.597656	NM_000251	MSH2	MutS homolog 2, colon cancer, nonpolyposis type 1 (E. coli)
E09	Hs.632177	NM_017458	MVP	Major vault protein
E10	Hs.202453	NM_002467	MYC	V-myc myelocytomatosis viral oncogene homolog (avian)
E11	Hs.2	NM_000015	NAT2	N-acetyltransferase 2 (arylamine N-acetyltransferase)
E12	Hs.654408	NM_003998	NFKB1	Nuclear factor of kappa light polypeptide gene enhancer in B-cells 1
F01	Hs.73090	NM_002502	NFKB2	Nuclear factor of kappa light polypeptide gene enhancer in B-cells 2 (p49/p100)
F02	Hs.9731	NM_002503	NFKBIB	Nuclear factor of kappa light polypeptide gene enhancer in B-cells inhibitor, beta
F03	Hs.458276	NM_004556	NFKBIE	Nuclear factor of kappa light polypeptide gene enhancer in B-cells inhibitor, epsilon
F04	Hs.103110	NM_005036	PPARA	Peroxisome proliferator-activated receptor alpha
F05	Hs.696032	NM_006238	PPARD	Peroxisome proliferator-activated receptor delta
F06	Hs.162646	NM_015869	PPARG	Peroxisome proliferator-activated receptor gamma
F07	Hs.654583	NM_000964	RARA	Retinoic acid receptor, alpha
F08	Hs.654490	NM_000965	RARB	Retinoic acid receptor, beta
F09	Hs.1497	NM_000966	RARG	Retinoic acid receptor, gamma
F10	Hs.408528	NM_000321	RB1	Retinoblastoma 1
F11	Hs.654402	NM_006509	RELB	V-rel reticuloendotheliosis viral oncogene homolog B
F12	Hs.590886	NM_002957	RXRA	Retinoid X receptor, alpha
G01	Hs.388034	NM_021976	RXRB	Retinoid X receptor, beta
G02	Hs.443914	NM_000454	SOD1	Superoxide dismutase 1, soluble
G03	Hs.479898	NM_005420	SULT1E1	Sulfotransferase family 1E, estrogen-preferring, member 1
G04	Hs.204044	NM_003839	TNFRSF11A	Tumor necrosis factor receptor superfamily, member 11a, NFKB activator
G05	Hs.472737	NM_003286	TOP1	Topoisomerase (DNA) I
G06	Hs.156346	NM_001067	TOP2A	Topoisomerase (DNA) II alpha 170kDa
G07	Hs.475733	NM_001068	TOP2B	Topoisomerase (DNA) II beta 180kDa
G08	Hs.654481	NM_000546	TP53	Tumor protein p53
G09	Hs.444319	NM_000367	TPMT	Thiopurine S-methyltransferase
G10	Hs.304249	NM_003358	UGCG	UDP-glucose ceramide glucosyltransferase
G11	Hs.654364	NM_000380	XPA	Xeroderma pigmentosum, complementation group A
G12	Hs.475538	NM_004628	XPC	Xeroderma pigmentosum, complementation group C
H01	Hs.520640	NM_001101	ACTB	Actin, beta
H02	Hs.534255	NM_004048	B2M	Beta-2-microglobulin
H03	Hs.592355	NM_002046	GAPDH	Glyceraldehyde-3-phosphate dehydrogenase
H04	Hs.412707	NM_000194	HPRT1	Hypoxanthine phosphoribosyltransferase 1
H05	Hs.546285	NM_001002	RPLP0	Ribosomal protein, large, P0
H06	N/A	SA_00105	HGDC	Human Genomic DNA Contamination
H07	N/A	SA_00104	RTC	Reverse Transcription Control
H08	N/A	SA_00104	RTC	Reverse Transcription Control
H09	N/A	SA_00104	RTC	Reverse Transcription Control
H10	N/A	SA_00103	PPC	Positive PCR Control
H11	N/A	SA_00103	PPC	Positive PCR Control
H12	N/A	SA_00103	PPC	Positive PCR Control
